# Effect of Meditative Movement on Affect and Flow in Qigong Practitioners

**DOI:** 10.3389/fpsyg.2019.02375

**Published:** 2019-10-22

**Authors:** Pasi Pölönen, Otto Lappi, Mari Tervaniemi

**Affiliations:** ^1^Cognitive Science, Department of Digital Humanities, University of Helsinki, Helsinki, Finland; ^2^Traffic Research Unit, Department of Digital Humanities, University of Helsinki, Helsinki, Finland; ^3^CICERO Learning, Faculty of Educational Sciences, University of Helsinki, Helsinki, Finland; ^4^Cognitive Brain Research Unit, Department of Psychology and Logopedics, Faculty of Medicine, University of Helsinki, Helsinki, Finland

**Keywords:** exercise, meditative movement, flow experience, affect, Qigong

## Abstract

Qigong is a Meditative Movement exercise that consists of mindful movements, regulation of breathing and attentional control. In this study we investigated whether Qigong practice might be associated with the affect and flow of its practitioners during the exercise. Although practitioners of Meditative Movement anecdotally describe flow-like experiences and strong effects on affect there are only a few empirical studies that focus on acute effects of Qigong practice on affect, and to our knowledge none on flow. Understanding these phenomena could shed new light on the interrelationship between body movement and the embodied mind. Self-reported affect and flow of qigong practitioners (*N* = 19) was probed in four qigong sessions, 1 week apart, each lasting about an hour. We used the PANAS (Positive And Negative Affect Schedule) to measure self-reported affect pre- and post-session. Additionally, open-ended questions were used to further inquire the specific quality of the post-session affect. Flow was measured using the Flow Short Scale, twice during each Qigong session and once after it. Our results confirm previous studies that Qigong practice shifts affect toward positive valence. Content analysis of the open-ended questions further revealed that the resulting experience can be described as restful, relaxed, happy, balanced, and clear. Although the lack of a control group/condition preclude drawing firm causal conclusions, our results indicate that Qigong practice produced flow already 20 min into the session, and that flow state intensified at 40 and 60 min. Future directions for studying affect and flow in meditative exercise are discussed.

## Introduction

The last few decades have seen an increasing interest in western culture toward oriental physical and mental practices such as meditation, Yoga and Martial Arts. Yoga can be considered part of mainstream culture with over 20 million practitioners in the United States alone (Schmalzl et al., 2015), and in the past years many different forms of “mindfulness” techniques have become increasingly popular. But while the positive effects on health and well-being from both physical exercise and meditative exercises are commonly accepted, forms of practice where these two are combined are still little researched and poorly understood (Payne and Crane-Godreau, 2013). Perhaps it is because of methodological challenges that meditative techniques based on executing large, coordinated movements – such as Qigong or Taijiquan – have had rather limited academic research done on them (Schmalzl et al., 2014).

This type of research would, however, be important for understanding human performance, well-being and the body–mind relationship from both applied and theoretical points of view. On the practical side it would be useful to have a clearer picture of the physiological and emotional effects of different types of exercise including those beyond traditional western forms. This would for example aid in assessing their potential benefits (or risks) for physical and mental health for particular individuals. Also, as performance in many fields (e.g., athletic performance) depends inseparably on both the physical and mental state of the performer, understanding the neural and psychological mechanisms involved in techniques explicitly combining body and mind could lead to novel methods of training and evaluation.

On a more fundamental level meditative techniques – and in particular movement-based meditative techniques involving the sensation and control of body movements – produce complex subjective experiences that may have unique features distinct those found in most everyday activities. These are interesting phenomena in their own right. This is particularly so if they can deepen our theoretical understanding emotion, as traditional emotion theories may need to be extended to incorporate new types of affect, or to describe the interplay of controlled body movement, somatic sensations and conscious attention in the generation of feelings and regulation of emotional state. Concerning this last point, “embodied mind” exercises also speak to a wider shift in cognitive science from paradigms that analyze cognitive processes and their sensory “inputs” and motor “outputs” separately, toward models of active perception (Ballard, 1991) and embodied cognition (Clark, 1999; Barsalou et al., 2003; Barsalou, 2008). In this framework, the motor system and the bodily mechanisms it controls directly contributes to cognitive processing, by actively shaping the sensory inputs and grounding explicit and abstract conceptual content in tacit, concrete sensorimotor processes. The upshot is that it is not possible to understand cognitive mechanisms in isolation of their bodily coupling with the body and the world.

Finally, on a phenomenological level our experience is, arguably, essentially that of an embodied agent in the world. We experience ourselves situated in a perpetual space with motor-specific affordances, we feel our emotions in the body, we frequently use bodily-spatial metaphors for describing social or abstract reality etc. Understanding how our sense of being-in-the-world arises from body and brain function – across the levels from detailed neurophysiology through basic psychological mechanisms to phenomenology of experience – would perhaps shed significant new light on some of the old mind–body problems.

Meditative movement practices, such as Yoga or Qigong combine specific movement patterns with mental techniques for reaching a meditative state of mind. The purpose of this study was to better understand the self-reported state of mind achieved in one such practice – Qigong – and in particular the possible elicitation of positive affect and flow-like experiences by the meditative movement training. Specifically, we investigated the effect of Qigong on the self-reported affective state measured by a standardly used self-report scale for positive and negative affect (Watson et al., 1988) and flow experience measured through a self-report questionnaire commonly used, e.g., to study flow in sports (Rheinberg et al., 2003). Also, to classify the language used by the participants to describe their affective state after the training we performed qualitative content analysis on verbal self-reports. These qualitative and exploratory methods are appropriate at this stage of research to shed light on the phenomenology of the mental state achieved in Qigong training.

As there are no well-established, precise definitions of meditative movement, positive affect or flow in the academic literature, we will next elaborate the meaning of these concepts in the context of this study.

The terminology and definitions used to describe meditative movement in the scientific literature vary (Payne and Crane-Godreau, 2013). Cardosoa et al. (2004) define *meditation* as practices with following features: (M1) Clearly defined techniques; (M2) Psychophysical relaxation, that leads to muscular relaxation; (M3) “logic relaxation” i.e., reduction of logical thought (need for evaluation, expectation, and explanation); (M4) The state is self-initiated and self-sustained; (M5) Attention is focused by anchoring concentration into a mental image, breathing, body state or some other target (Note: there exist two meditation styles, with respect to focusing of attention and awareness: focused attention and open awareness. M5 only applies to focused attention style meditation). Payne and Crane-Godreau (2013) use the term Meditative Movement for forms of physical exercise where such meditative attention is directed toward bodily sensations, including proprioceptive, interoceptive, and kinesthetic sensations. They include traditional Chinese methods such as Qigong and Taijiquan, as well as certain styles of Yoga and other oriental practices such as Aikido and Sufi Dance, but also certain western somatic practice forms such as Alexander Technique and Feldenkrais. On the other hand, in their study on Qigong, Johansson and Hassmén (2013) use the term mind-body therapies for Qigong, Taijiquan, meditation and Yoga. Schmalzl et al. (2014) use the term Movement-based embodied contemplative practices for traditional oriental forms such as Yoga, Qigong, Taijiquan, and modern western somatic methods such as the Alexander technique and Feldenkrais-method.

Larkey et al. (2009) propose *Meditative Movement* as designation for a category of forms of exercise, such as Taijiquan and Qigong, that combine (MM1) some form of movement or body positioning, (MM2) a focus on breathing, and (MM3) a cleared or calm state of mind with a goal of (MM4) deep states of relaxation. They consider meditative movement a class mind–body or body–mind exercises that is different from traditional (western) exercise forms in that the focus is on bodily sensations and breathing, and achievement of a state of relaxation (This is in contrast to focus on external targets and performance goals, and achieving certain performance measures.) Meditative Movement in this sense can be considered as a superordinate class of exercise forms analogous to aerobic exercise.

We adopt the term Meditative Movement, and use it in the sense proposed by Larkey et al. (2009). We leave at the moment open whether the lists (M1–M5 and MM1–MM4) should be augmented to exclude sports with, arguably, similar features but very different techniques to traditional meditation – such as long-distance running or free diving – or whether indeed these fields should be considered as forms of meditative movement. Even in this case we take it, however, that under any reasonable further restrictions into the definition, traditional movement based meditative techniques are to be included in the definition of Meditative Movement. This includes Qigong which is a meditative movement form comprising of movements, static poses, regulation of breathing as well as various cognitive techniques such as somatic awareness, mental imagery, and focusing of attention (Cohen, 1999; Jahnke et al., 2010; Klein et al., 2016; Osypiuk et al., 2018). Overall the general purpose of Qigong practice is to increase vitality, balance circulation and to harmonize body–mind relationship.

*Affect*, emotion and feeling are used, sometimes interchangeably, to designate those episodes of our experience that have some emotional component. Emotion is often considered to comprise of physiological (e.g., hormonal), bodily (e.g., facial expression, pose) and mental (e.g., attentional or memory-retrieval) components, not all of which are accessible to consciousness. That is, there are processes involved in an emotion other than the *feelings* arising from emotional processes which we can report.

Here, we use the term affect in a broad sense. We consider the term affect to denote episodes of emotional life with a time scale of some minutes, and which can be brought into awareness and explicitly reported (This in contrast to, e.g., mood which can persist for hours or days.) Affect can be investigated by both self-report measures as well as psychophysiology. Only self-report measures were used in this study. However, we do not pre-theoretically commit to the notion that affect always has, by definition, a positive or negative *valence*. Although some theorists define affect thus, we consider it would be pre-judging the issue to assume all affect must contain such evaluative component (This is particularly pertinent when considering meditative states of mind, where a neutral affect, yet distinct from boredom, tiredness or other typical forms of “flat affect” might be prevalent.) Also, we do not commit to the notion that the affective episodes in Meditative Movement should be categorizable into traditional *basic emotions* (as in categorical emotion theories), or constructed from arousal, valence (and possibly a “cognitive” evaluation). Meditative movement elicits complex emotional-cognitive-bodily experiences, and to what extent they conform to, and are captured by traditional emotion-concepts is an open issue. Therefore, we consider “affect” to be the most neutral term to adopt.

Many emotion theories regard affect as “the experiential component of all valenced (i.e., ‘good’ or ‘bad’) responses, including emotions and moods” (Scherer, 1984; Lazarus, 1991; Frijda, 1993; Gross, 1998, 1999) in Ekkekakis and Petruzzello (2000, p. 77). We do not, on two counts. Firstly, we do not restrict the term affect to the consciously reportable feeling, and in particular a good/bad evaluation (valence). There are two reasons. One is that, affective neuroscience or models of affective processes can involve aspects of affective states that are not reportable, and affect can be studied in non-human species where the issue of reportability is problematic. The other is that, especially in regard to meditation, we think it is not a good idea to paint oneself into a theoretical corner by defining affect as including an evaluative component of positive vs. negative. Leaving the definition open means that meditative states can conceivably involve affect, even powerful affect, in the absence of a good/bad evaluation. Secondly: we do distinguish affect and mood – by duration, although there need not be a hard cut-off for when an affective episode has lasted long enough to become mood. This is more out of convenience, rather than a conviction of differences in underlying mechanisms. However, in terms of response to psychological or pharmacological manipulations, and experimental operationalization, there is a difference in rapidly shifting affective episodes and more persisting emotional-motivational state, the latter of which we would call mood.

*Flow* (Csikszentmihalyi, 1975; Nakamura and Csikszentmihalyi, 2002) is a complex psychological construct, developed to describe phenomenologically the enjoyment people get from activities that they engage with, often with no apparent external reason or incentive. The flow state is sometimes achieved during challenging task performance (“deep flow”) or more mundane activities like watching television (“microflow episodes”). It is characterized by the following features: (F1) total immersion and attentional focus in what one is doing; (F2) merging of action and awareness (being ‘one’ with the task and/or the environment); (F3) loss of reflective self-consciousness, and/or a sense of effortlessness and automaticity; (F4) a sense of control and confidence; (F5) an experience of the activity as highly enjoyable; (F6) sustained motivation (if given the choice, you choose to continue doing the task rather than to discontinue); (F7) a distortion of temporal experience (time may seem to go slower or faster than normal). Tasks which elicit flow have an autotelic quality, i.e., people will want to do Flow-producing activities again and again, regardless of external reward. Although the flow state is a multidimensional phenomenon as described above [F1–F7 combines *cognitive* (e.g., attention, automaticity), *affective* (enjoyment), *behavioral* (self-reinforcing or “autotelic”) aspects as well as subjective experience (emotional quality of enjoyment, awareness of self, time, and space)] typically flow is studied through self-report questionnaires. Here we, too, concern ourselves with the self-reported *flow experience*. Note that enjoyment (F5) does not necessary imply pleasure or positive hedonic experience or positive valence – consider, e.g., mountaineering in challenging conditions, or a long-distance runner enduring pain.

To better understand the complex experiences during Qigong practice, and to operationally disentangle flow and affective valence, we used both standard flow and standard affective valence questionnaires. Previous work on Qigong has shown Qigong training to have an overall positive effect on the affective state of participants (e.g., Payne and Crane-Godreau, 2013; acute effects of single Qigong sessions, e.g., Lee et al., 2004; Johansson et al., 2008; Johansson and Hassmén, 2013; for reviews see Jahnke et al., 2010; Wang et al., 2010; Chi et al., 2013; Wang et al., 2013). We set out to study acute affective effects of Qigong training and the presence of Flow in a longitudinal design with four sessions. Previous work using self-report questionnaires had indicated positive affective state is elicited by many forms of exercise, including Meditative Movement, and we set to replicate this finding. To the best of our knowledge, Qigong has not been previously investigated with standard Flow self-report instruments.

Additionally, we wanted to explore further into the *quality* of the experienced affect, which may go beyond simply positive vs. negative valence or the presence/absence of flow. For this purpose we decided to collect open ended self-report data of the participants’ descriptions of their emotional experience in their own words for qualitative content analysis.

In sum: by combining widely used measuring tools with open self-reports gives us a more nuanced picture of the complex affective experience of participants. This is a similar approach than what, e.g., Rose and Parfitt (2007) and Johansson and Hassmén (2013) have used. While positive affect during Qigong has been reported in the literature, there are to the best of our knowledge no studies relating this form of Meditative Movement practice to flow. Given that there are parallels between the elicit conditions and experience of flow and the meditative state, we expected qigong exercises to be conducive to the experience and to probe this hypothesis we also administered a widely used questionnaire for self-reported flow.

## Materials and Methods

**FIGURE 1:**
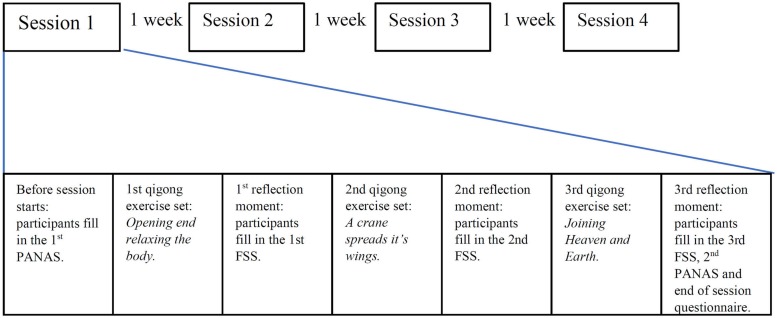
The longitudinal design of the study, and the structure of each Qigong session.

The experiment had a longitudinal design where the participants (*N* = 19) participated in four weekly 90 min group training sessions (see Figure 1). In each session three different Qigong exercise sets were performed. A 5 min reflection period followed each exercise set, during which the participants were encouraged to probe their own mental state introspectively for a few minutes, after which they proceeded to fill in the Flow Short Scale (FSS) self-report questionnaire. Participants also filled in the Positive And Negative Affect Schedule (PANAS) self-report questionnaire before and after each session. These responses were used in the statistical analyses. At the end of sessions 2,3 and 4 the participants filled in an open verbal-self report of their affective states. This data was analyzed using qualitative content analysis.

### Participants

The participants (7F, 14M) were recruited from among the Finnish Qigong community by email. There were no explicit exclusion criteria, but all participants were healthy adult meditative movement practitioners who had experience with this type of exercise. Following Jahnke’s (review Jahnke et al., 2010) recommendation using Qigong as main category all previous training experience whether it was any form of Qigong or any form of Chinese Internal Martial Arts such as Taijiquan or Yiquan, was categorized as Qigong experience.

Participants gave informed written consent to partake in the experiment and the use of the data for scientific purposes in accordance with the Declaration of Helsinki. The data collection procedure does not contain aspects that according to the Finnish Advisory Board on Research Integrity^1^ would require ethical pre-evaluation. The data were collected as part of an undergraduate thesis project, which is exempt from ethical pre-evaluation to be conducted by the University ethical review board. However, we have submitted the procedure to the University of Helsinki Ethical review board in humanities and social and behavioral sciences for a series of follow-up experiments with identical procedure but using slightly different questionnaires. It has been declared ethically acceptable (Statement 27/2019).

Background information was collected with a form that asked for their age, sex, and training experience. The participants were aware they were participating in a scientific study, but were naïve with respect to the research questions. The participants were informed that it was desirable that they participate in all four sessions, but that missing one session would not invalidate their data.

Nine participants took part in all four sessions, and 10 in 3 sessions. Two participants only took part in two sessions and their data was disregarded from the study. The age of the participants that took part in at least three sessions (*N* = 19, 7F 12M) ranged from 26 to 78 year (mean: 50 year, *SD*: 15 year). Prior experience in Qigong ranged from 3 months to 20 year (mean: 6.5 year, *SD:* 6.7 year). Weekly training ranged from 0 to 8 sessions (mean: 3.5, *SD:* 2.7). It was not controlled what kinds of practices participants did between sessions (if any).

### Session Protocol

During each Qigong session, the participants performed three Qigong exercise sets, each lasting about 20 min. This was done in a group, led by the first author (PP), who has taught Qigong and Chinese Internal Martial Arts for more that 20 years. The exercises were based on the Dynamis-qigong style developed by him. Filling out the questionnaires took altogether about 30 min. The entire session lasted about 1.5 h.

The aim was to make all the sessions as similar to one another as possible in terms of ordering of the exercises and the duration of the phases, so that longitudinal effects would not be introduced by differences in session content, and also to minimize the effects of a participant missing one session. Thus, each session followed the same pattern, as follows:

(1) Beginning of session. Participants fill in the PANAS self-report questionnaire.
(2) First Qigong exercise: “Body opening and relaxing exercises,” comprising of a variety of slowly performed waving, shaking, stretching and circling movements, while focusing attention to different bodily sensations (video link: https://www.youtube.com/watch?v=2lYm_z8SlUk).
(3) First reflection moment. Participants fill in the FSS self-report questionnaire.
(4) Second Qigong exercise: “A crane spreads its wings,” combining motion, breathing, and directing attention to harmonizing movement and breathing (video link: https://www.youtube.com/watch?time_continue=37&v=a_BleyXFsig).
(5) Second reflection moment. Participants fill in the FSS.
(6) Third Qigong exercise: “Joining heaven and earth,” where movement, breathing and different mental images, such as imagining warmth or light flowing through the body, in coordination with movement and breathing, are integrated using attentional focus (video link: https://www.youtube.com/watch?v=wqb66SuL-HE).
(7) Third reflection period. Participants fill in the FSS, PANAS, and end-of-session debriefing questionnaire. End of session.

This structure follows the common training methodology in Qigong of moving from temporally extended and “external” large scale movements to ever smaller and subtler “inner movement,” which in the tradition is regarded as the most powerful but also demanding form of exercise (Cohen, 1999; Schmalzl et al., 2014). Also, a consideration in developing the protocol was that the FSS questionnaires between exercises should not disrupt the state of meditative focus, and they were hence introduced into the sessions as part of a “reflection moment.”

### Instruments

The PANAS and FSS self-report questionnaires were used to assess affective effects and the elicitation of flow, respectively.

Positive And Negative Affect Schedule is a paper and pencil questionnaire designed to measure positive and negative affect (valence). It consists of twenty items, 10 with adjectives describing positive and 10 describing negative affect. Watson et al. (1988) demonstrated internal consistency for the PANAS ranged between 0.86 and 0.90 for positive affect and 0.84–0.87 for negative affect. Test–retest reliability for the PANAS (1 week) were reported as 0.79 for positive affect and 0.81 for negative affect (Watson et al., 1988).

Pre-session, the participants answered the question “How have you felt during the past few hours overall” and after the session “How do you feel right now” on a five-step Likert scale anchored at 1 = not at all or very little to 5 = very much. Total PANAS score was calculated by subtracting the sum of negative items from the sum of positive items (i.e., sign of the deviation from zero indicates the overall positive valence). Subtracting the pre-session total PANAS score from the post-session PANAS score yielded a measure for the change in affect during the session.

Flow Short Scale is a paper and pencil questionnaire designed to measure the experience of flow (Rheinberg et al., 2003). It consists of 10 Likert scale questions anchored at 1 = Not at all and 7 = Very much. There are two scales, fluency of performance (six questions), and absorption by activity (four questions). The fluency and absorption scores were averaged, and then summed to give the total flow score as per Rheinberg et al. (2003). Cronbach alpha for fluency and absorption was (0.92). There are no standard Finnish translations of PANAS or FSS. The version used here is based on an existing PANAS translation which was slightly modified by PP and MT. The FSS used was the translation used in Cowley et al. (2019) with some slight edits.

### Post-session Debriefing

Additionally, at the end of each session the participants filled in a debriefing questionnaire, containing further questions about the achievement of flow, open-ended questions for self-reporting their current affective state, as well as questions pertaining to the organization of the experiment. At the end of each four sessions, the participants filled a self-report questionnaire asking them about the session as a whole. There were three items based on the FSS perceived importance scale, another three based on the FSS skills and demands scale (both answered 1–7 Likert scale anchored 1 = not at all, 4 = partly, 7 = very much), and four questions concerning the practical arrangements of the experiment.

Note that unlike Rheinberg et al. (2003), the three FSS perceived importance scale items and three FSS skills and demands items were only presented to the participants at the end of the session, and the participants were instructed to answer on the basis of how they experienced the session as a whole. The main reason was to keep the breaks between the Qigong exercise sets short, in order not to disrupt the meditative frame of mind.

Positive And Negative Affect Schedule scores measure total positive vs. negative affective state (valence), but do not give further insight into the quality of the experience. For this purpose, the participants were asked (at the end of sessions 2–4) to describe their feelings by answering in their own words “What words or sentences would best describe your emotional state right now after the session?”

Also, participants were asked whether they felt the reflection periods and questionnaire-filling were disruptive to the maintenance of a meditative focus. The aim was to make the self-report questionnaires as naturally a part of the normal course of a Qigong session as possible, and this question was used to evaluate how well this succeeded. The final item in the debriefing questionnaire was open space, where participants could write whatever comment they had (if any) concerning the session.

### Content Analysis of the Open-Ended Responses

The participants gave written open-ended answers to the question “what words or sentences would best describe your emotional state right now after the session?” Words/expressions describing the respondent’s affective state in the open-ended responses were classified into categories by an iterative process as follows. The first author (PP) first went through all the material, identifying 64 meaning units, with a total of 117 occurrences, and grouping them into classes of units judged to have similar meanings based on synonymy using a thesaurus. The principle was to classify each meaning unit to exactly one class.

From this, nine classes emerged, labeled peaceful, cheerful, energized, relaxed, drowsy, balanced, tense, present, ambiguous (word meaning was unclear). Based on these labels alone, the second author (OL) then independently classified the meaning units into those nine classes. Discrepancies between the authors’ classifications were identified, and the differences in interpretation discussed. On the basis of this, the category labels were slightly revised, and each category was given a short verbal description of the intended interpretation of the category label.

This augmented categorization was then given to three naive classifiers, who were not involved in the preceding discussions, nor experienced in Meditative Movement (but did have some experience of survey and self-report questionnaire research). They were independently given written instructions to classify each word into exact one category, and use the category “unclassified” if the meaning unit does not clearly fit any of the other categories. Based on the naive respondents’ classifications, and feedback from debriefing them on how they interpreted the category labels and descriptions, the categorization was refined still, yielding the nine classes used to categorize the respondents self-reported affective state in this study. Finally, this classification scheme and instructions was given to 14 classifiers who independently classified each word to one of the nine categories.

English translations for the category labels with examples, and the class descriptions provided to the classifiers are given in Table 1.

**TABLE 1:** The nine categories of affective quality that emerged from the analysis of the open-ended self-report questionnaires.

**Class number**	**Class name**	**Class description**
1	Restful	Sedation of body/mind; relaxed body, feeling drowsy (note: tiredness nevertheless a separate class). *Examples: restful, relaxed*
2	Energized	Lightness and ease of bodily movement, sense of empowerment in body or mind (opposite of drowsy). *Examples: vital, energized*
3	Tired	Heaviness/slowness of physical movement, sense of fatigue in mind/body (opposite of energized). *Examples: slowed, tired*
4	Lucid, clear	Mind is calm and clear “like a calm sea/lake.” *Examples: cleared, clear, and calm*
5	Balanced	Words related to balance and stability. *Examples: balanced, tranquility, and balance*
6	Positive affect	Any positively colored “mental” (rather than physical) experience not fitting the above categories. *Examples: cheerful, happy*
7	Negative affect	Opposite of positive affect. *Examples: painful, tense*
8	Present	Conscious, mental awareness (mindfulness). *Examples: awake, present*
9	Other (unclassified)	Note: if the word does not CLEARLY fit any of the above classes, classify it here. *Example: healed, a bit empty*

The classifications of the meaning units by the 14 naïve classifiers was used to calculate each meaning unit a semantic consensus rating as to how well the meaning unit conveys the meaning of the category. This consensus rating represents each word occurring in the responses as a vector of nine dimensions, each ranging from 0 (i.e., no naive classifier classified the word as belonging to category) to 1 (i.e., all classifiers classify the word as belonging to the category), summing to one (so for example, if all classifiers considered a particular word to belong to class 3, the word would be represented as [0,0,1,0,0,0,0,0,0], for another word the consensus might be marginally less, with one respondent classifying the word into class nine, giving [0,0,0.929,0,0,0,0,0,0.071], another word might be more ambiguous still, yielding, e.g., [0.143,0,0.716,0,0,0,0.71,0,0.071] etc.). The semantic consensus ratings of all meaning units are given in Supplementary Table S1.

## Results

This section first describes the quantitative results of the effects of the Qigong exercises on self-reported affect and flow-experience, as measured by the PANAS and FSS scales. Then, the qualitative results of the content analysis of open verbal questions are given.

### Statistical Analysis of PANAS and FSS

The data was analyzed using IBM SPSS 25.0. Preliminary analyses were done to plan the final statistical analysis. Namely, the participants were grouped according to whether they had prior experience specifically of the Dynamis-qigong style or not. No statistically significant differences in PANAS or FSS results were found using Mann–Whitney test (PANAS: *Z* = −0.575, *p* = 0.565, FSS: *Z* = −0.868, *p* = 0.391) and in the rest of the analyses all participants were treated as one group. Group comparison analysis were also performed with sex, age, frequency of weekly practice and years of experience with PANAS and FSS scores, but no statistically significant effects were found. Therefore, in the statistical analyses reported below all participants are treated as a single group, and sex, age or experience effects were not investigated further.

#### PANAS Results

**FIGURE 2:**
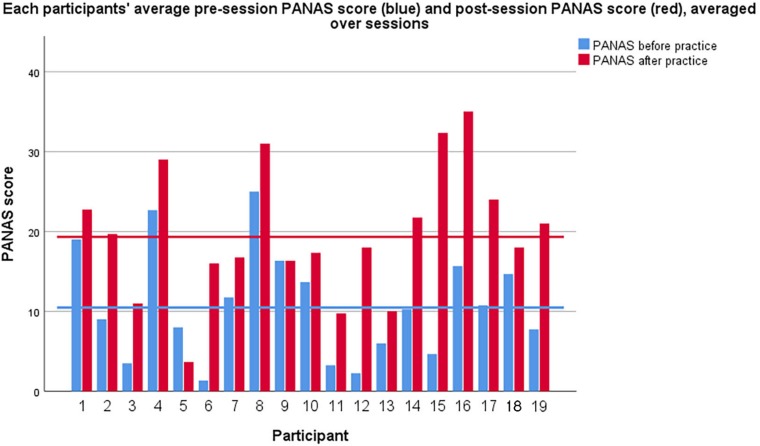
Each participants’ pre-session (blue) and post-session (red) PANAS score averaged across sessions. Horizontal lines indicate group pre- and post-means. X axis: Individual participants. Y axis PANAS score.

**FIGURE 3:**
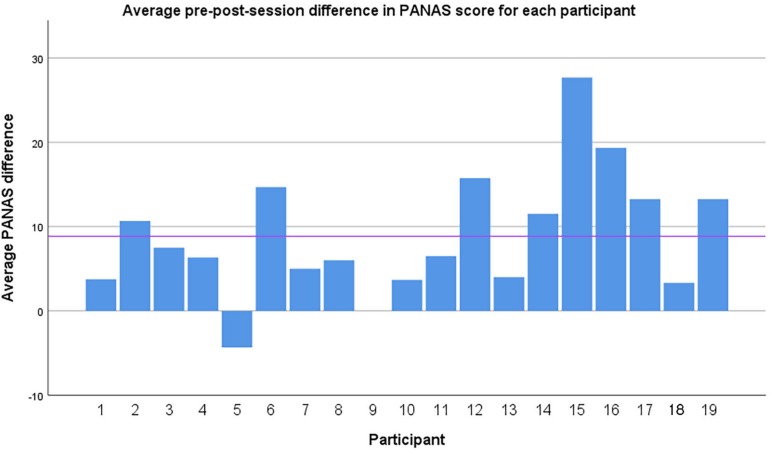
Mean pre-post-session change in PANAS score for each individual participant. Horizontal line indicates group mean. Note: participant 9 has identical pre- and post-PANAS scores. X axis: Individual participants. Y axis: PANAS score.

Affective state was measured by PANAS at the beginning and end of the session (Figure 2). There was an increase in positive affect and decrease in negative affect during sessions (Table 2). Total change in PANAS score can be seen in Figure 3. The range of the PANAS scale is [−40, 40], and comparing the pre- and post-session scores within subjects the average change is + 8.85 (over 10% of the range), which may be considered substantial. Both negative and positive affect changes reached significance on the non-parametric Wilcoxon test (Table 3). The results support the hypothesis that, as found in prior studies, qigong exercises can induce a more positively valenced affective state. A Friedman test revealed no significant differences in PANAS scores between sessions 1–4 [df (3), *p* = 0.95].

**TABLE 2:** Pre- and post-session PANAS scores, averaged across the four weekly sessions.

	**Mean**	***SD***
PANAS pre: negative	15.20	6.29
PANAS post: negative	10.77	2.64
PANAS pre: positive	25.67	8.51
PANAS post: positive	30.09	9.71
PANAS pre: total	10.47	9.95
PANAS post: total	19.32	9.36

**TABLE 3:** Wilcoxon test on PANAS scores.

	**Negative affect change**	**Positive affect change**	**Total affect change**
Z	–5.98	–4.22	–5.57
Asymp. Sig. (two-tailed)	<0.001	<0.001	<0.001

#### FSS Results

A match between skill and task demand is generally considered a precondition of flow. This was probed by explicitly asking the participants to rate the task difficulty of the session on a scale of 1–7, with anchors 1 = “too low,” 4 = “just right,” and 7 = “too high.” As shown in Table 4, the majority (86.4%) of the answers indicated an appropriate level of task difficulty, which a good fit between task demand and capability.

**TABLE 4:** Match between skill and task demand.

**The degree of difficulty**	**1**	**2**	**3**	**4**	**5**	**6**	**7**	**Total**
**of this session was**	**too low**			**just right**			**too high**	
*N*	1	3	5	57	0	0	0	66
%	1.5	4.5	7.6	86.4	0	0	0	100

**FIGURE 4:**
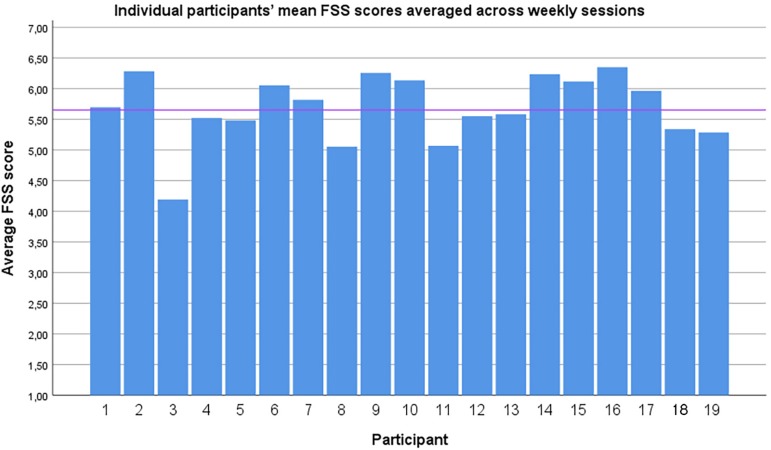
Individual participants’ mean FSS scores averaged across weekly sessions. Horizontal line indicates group average. X axis: Individual participants. Y axis FSS score.

**FIGURE 5:**
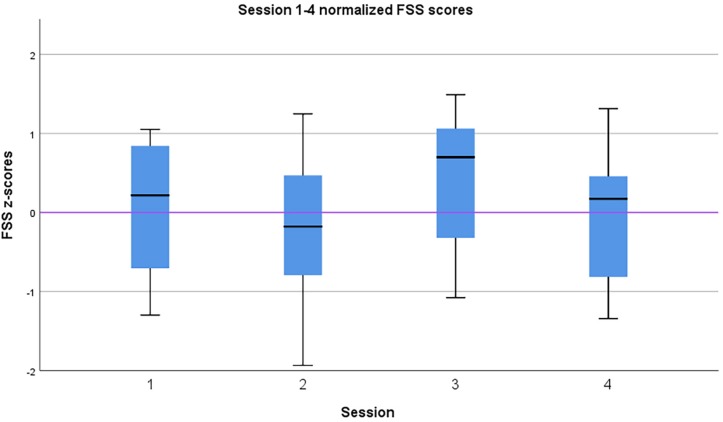
Session 1–4 normalized FSS scores. X axis: sessions 1–4. Y axis: normalized FSS score.

The experience of flow itself was measured with the FSS. The average flow (averaged across participants and sessions) was mean = 5.66, *SD* = 0.63 (see Figure 4). The scale range is [1.00, 7.00]. Figure 5 shows a box plot of Z-normalized flow scores of the four sessions. The FSS scores of the qigong exercise sets 1–3 (averaged over the four weekly sessions) are shown in Table 5. The FSS scores of the four weekly sessions (averaged across exercise sets) are shown in Table 6.

**TABLE 5:** Flow Short Scale scores after exercise sets 1–3, averaged across sessions.

	***N***	**Minimum**	**Maximum**	**Mean**	***SD***
1^st^ exercise set: *Opening and relaxing the body*	66	3.63	6.75	5.45	0.77
2^nd^ exercise set: *A crane spreads it’s wings*	66	3.33	6.79	5.70	0.70
3^rd^ exercise set: *Joining Heaven and Earth*	66	2.83	6.83	5.82	0.81

**TABLE 6:** FSS scores of sessions 1–4.

	***N***	**Minimum**	**Maximum**	**Mean**	***SD***
Session 1 FSS	19	4.83	6.32	5.69	0.51
Session 2 FSS	19	4.43	6.44	5.54	0.57
Session 3 FSS	19	4.08	6.60	5.84	0.65
Session 4 FSS	9	3.42	6.49	5.43	0.90

Friedman test revealed the mean FSS scores of the exercise sets 1–3 different significantly df(2), *p* < 0.001. Pairwise comparison with Wilcoxon test indicate a difference between the first and second exercise sets (*Z* = −3.027, *p* = 0.002) and between first and third exercise sets (*Z* = −3.743, *p* < 0.001). I.e., the second and third exercise sets FSS scores were statistically significantly higher than those of the first.

### Content Analysis

**FIGURE 6:**
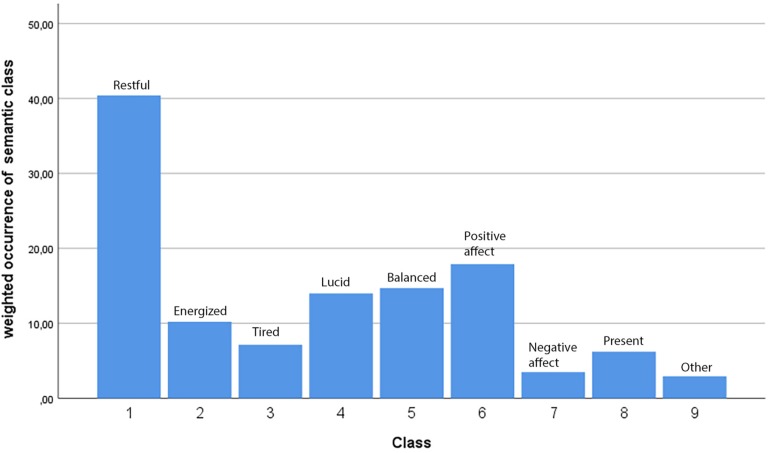
Weighted occurrence of each semantic class in the open response data.

Our use of semantic classification of each meaning unit into nine categories of experience allowed us to study the occurrence of each *category* in the response data, as opposed to merely counting frequencies of individual *meaning units* (most of which were used only once). For this, we simply multiplied the frequency of occurrence of each meaning unit with the weightings given by that meaning unit’s semantic consensus ratings, yielding a table of weighted occurrences of each category. In other words, the participants’ use of a particular meaning unit contributes to the occurrence of each semantic class through the scalar product of word frequency and semantic consensus vector. The weighted occurrence of the class is then simply the component-wise sum of each meaning unit’s weighted contribution to the class. The meaning unit frequencies and weighted contributions to class occurrence are given in Supplementary Table S1. The weighted occurrence of each semantic class is shown in Figure 6.

By far the highest occurring semantic category was 1 Restful category followed by 6, Positive affect and 5, Balanced and 4 Lucid, clear. These four categories covered 74.36% of our classification. Interestingly, both category 2 Energized, and its polar opposite 3 Tired, were about equally represented. 8 Presence/mindfulness, 7 Negative affect and 9 Unclassified were only weakly expressed in the material. The predominant affective states that qigong practitioners reported in our study can be summarized as feeling restful, relaxed, happy, balanced and clear.

### Other Results

Participants were asked whether they felt the reflection periods and questionnaire-filling were disruptive to the maintenance of a meditative focus. Out of 60 answers only three subjects reported disruptions (9.67% of all answers). Majority of subjects (67.74% of all answers) felt that reflection periods were positive and enhancing. The rest of the answers (22.58%) were neutral.

## Discussion

In this study we investigated whether Qigong practice might be associated with the affect and flow of its practitioners during the exercise. Using a repeated measurements paradigm and both standard questionnaire and open-ended self-report methods, we were able to determine the effects of Qigong on affect and the experience of flow. Affective valence changed toward positive, as measured with PANAS pre- and post- session, in a timeframe of about 1 h. [It is of course possible that affective change happens much faster, as suggested study done by Johansson and Hassmén (2013), affective change happened in 20 min.] Our open questions suggest most prominent affective state experienced during Qigong can be characterized as restful, relaxed, happy, balanced and clear. Note that for the most part these affective qualities did not have much overlap with PANAS items. We interpret that there is room to develop a more fine-grained picture of the possibly multidimensional affective changes in meditative movement.

Flow was measured with FSS, and measurements suggest that flow was achieved by the first 20 min measurement point and it increased further in 40 and 60 min measurement points. Since there was no counterbalancing of the three Qigong exercise sets we cannot say if this increase is as result of different types Qigong exercises, or if flow experience increased simply as a function of time. In the open-ended responses, some subjects described their Qigong experience in a way that resembles the way that flow is described, such as: “Today I felt a bit tired, although also concentrated and calm. Training session woke me up and brought very enjoyable concentration in to the body, even flow-like experience where my body moved automatically.” These results are accordance with Csikszentmihalyi’s view that oriental meditative movement arts and Martial Arts are designed to produce flow (Csikszentmihalyi, 1990).

The amount of previous Meditative Movement practise, sex or age did not appear to affect PANAS or FSS results in our small sample, and so we did not investigate these in more detail. All participants of this study had at least several months of previous Meditative Movement experience and it seems that was enough time to obtain adequate skill to gain positive effects of this practice.

There was lot of variance between different subjects and between subjects in different sessions in regards PANAS and FSS.

### Limitations and Future Directions

Meditative Movement is as-yet quite little studied, and the appropriate methods to understand it are still in developmental stages. Two questionnaires from more established lines of research (affect and flow) were used here for better comparability into existing research. It is likely that gleaning more accurate and nuanced information about Meditative Movement will require dedicated self-report instruments that take into account the special features of Meditative Movement as an exercise form. Such methods are indeed under development, such as the Meditative Movement Inventory (Larkey et al., 2009). This is a 17-item questionnaire aiming to capture in descriptions different aspects of meditative movement, such as the meditative state of mind, breathing, flow of movement and affective quality.

Cross-cultural commensurability is always an issue in questionnaire based research, due to linguistic and cultural differences that need to be reconciled in translating questionnaire items. There are no generally accepted Finnish translations for PANAS and FSS. The version of PANAS used here was an existing translation. Some items such as “excited” and “enthusiastic” have subtle differences that are difficult to convey in standard Finnish. It is however unlikely that this would have seriously affected the results. The vocabulary used in PANAS is generic and measures wide range of emotional states. In our open-ended questionnaire only in four instances out of 64 classified answers the same words as in PANAS questionnaire were used (alert once, nervous once, and active twice). Thus, our analysis of open-ended self-report questions allow us to capture more subtle differences in the emotional state meditative movement was experienced in our participants. A dedicated questionnaire designed specifically for self-report of affective states in meditative movement might have higher validity and reliability, and could prove useful for probing in more detail the emotional effects of other forms of exercise as well.

Regarding FSS questionnaire, it is not clear how well two of the perceived importance items “I must not make any mistakes here” and “I am worried about failing” fit Qigong – especially the Dynamis-qigong approach used here. The participants were explicitly told before the session to find their individual way to perform the exercises, so there was no normative way to “fail” or to define “error,” as such.

Flow was probed with FSS after three 20-min Qigong exercise sets. Such after-the-fact reports cannot tell about the fluctuation of flow during the exercises, or, indeed, whether the emotional experience corresponding to the items was at all present during performance or only emerged after completion of the exercise. This is a fundamental limitation of all after-the-fact self-reporting. It is particularly vexing in the case of studying flow, because flow is by definition a state of reduced self-consciousness and therefore external probes to report one’s flow might themselves disrupt the very phenomenon. After-the-fact vs. concurrent report of flow is an issue that needs serious methodological work in the field of flow research.

The results suggest that already after the first exercise, a state of flow was achieved. It would be interesting both from a practical and theoretical points of view to know how quickly experienced practitioners of Meditative Movement can “enter into flow.”

One limitation of the study is that the majority (89%) of the participants had previously participated in Meditative Movement training led by the first author. Thus, if the participants interpreted the aim of the sessions to induce positive feelings or elicit flow, experimenter bias could be introduced to the PANAS and FSS scores. Care was taken to present all oral and written instructions in as neutral and non-leading way as possible, but ultimately there is no way to determine the magnitude of the issue, or ascertain how well it was mitigated. In meditation research this problem of bias might be seen as especially worrying. However, in their Meta-Analysis on meditation Sedlmeier et al. (2012) did not find experimenter bias, participants’ expectation or personal relationship effects to have a strong effect on results.

The three different Qigong exercise sets were repeated in the same order in each session in order to follow the common Qigong progression from large- to small-scale movement. This introduces systematic within session differences in exercise type before each FSS report. However, because this type of ordering corresponds to the “natural” ordering of the exercises for Qigong, it was judged that ecological validity of the session structure was more important than counterbalancing the exercise types, which would have allowed us to probe the question of whether specific individual types of exercises are particularly conducive to flow, or associated with positive affect.

More research is also needed to understand the effects of Meditative Movement on flow, such as how fast can an experienced practitioner reach a flow-state. Another interesting avenue would be to combine flow questionnaires with psychophysiological measurements such as EEG (Electroencephalography), HRV (Heart rate variability), or EDA (Electrodermal activity). However, a problem with psychophysiological measurements are their sensitivity to movement-induced errors in signal, which can be major problem in Meditative Movement research. Still, wearable sensor technology is rapidly evolving that might alleviate this problem. Also, there exist several forms of Meditative Movement practices were physical movement is very small, such as Yiquan (“intention boxing”) were the practitioner stands still for long periods of time in certain postures (such as “hugging the tree” -posture) and uses mental (motor) imagery that produces only small micromovements in the body. Since the movements in this kind of practice are very small, recording psychophysiological signals would likely be easier than in most forms of Meditative Movement (or exercise generally), in terms of avoiding signal corruption from motor artifacts.

Also, to get a clearer view of the underlying principles specific to Meditative Movement, its effects on flow and affect should be systematically compared to forms of sitting meditation, regular relaxation techniques, or regular physical exercise. Later on, systematic comparative studies even with other activities known to be associated with mood regulation and flow experience, such as dance or music listening or sports, should be conducted. Large-scale empirical efforts along those lines would highlight commonalities and differences between different meditation methods and, in a broader framework, between different types of mood regulation and management of stress. Yet, it needs to be acknowledged that such controlled experiments remain a challenge for future studies, as it is first necessary to identify the (theoretically) most significant aspects of Meditative Movement/qigong in order to determine relevant independent variables and controls. Additionally, it is necessary, to identify the (theoretically) most significant effects in order to determine meaningful dependent variables. In parallel, as already pointed out earlier, we need to work to determine the most sensitive and reliable methodological choices. Thus, at the present state of research on psychological concomitants of meditative movement, in our judgment, exploratory groundwork like the work presented here is an important starting point.

## Conclusion

Meditative movement practices combine specific movement patterns, breathing and mental attention focusing techniques to achieve cognitive and emotional changes in the human, understanding of which could shed new light on the interrelationship between the body and the embodied mind. Although self-report data has limitations, and can usually only be collected after-the-fact, they are nevertheless the only way more subtle questions about the quality of affective experience or the phenomenon of Flow experience can be experimentally posed. Established questionnaires and more qualitative techniques of analyzing the way participants describe their inner feelings in their own words can hopefully pave the way for the development of psychometrically validated questionnaires designed specifically for the study of Meditative Movement, and even psychophysiological measurements. Such complementary techniques should allow us to better understand the mental and physical changes produced by sustained practice of Meditative Movement, and, further, to lead to theoretical advancements in understanding the complex interplay of body movement, breathing, and attention in the generation of human experience.

## Data Availability Statement

The datasets analyzed for this study can be found on figshare: https://doi.org/10.6084/m9.figshare.9638024.v1.

## Ethics Statement

Ethical review and approval was not required for the study on human participants in accordance with the local legislation and institutional requirements. The patients/participants provided their written informed consent to participate in this study.

## Author Contributions

PP conceived the study and ran the experiment. PP, OL, and MT designed the experiment, wrote the manuscript, first draft, and approved the manuscript for submission. PP and OL analyzed the data.

## Conflict of Interest

PP has been teaching professionally different Meditative Movement methods (including Dynamis-qigong that were used in this study) over 20 years, and the majority of subjects had participated at least once in his classes. The remaining authors declare that the research was conducted in the absence of any commercial or financial relationships that could be construed as a potential conflict of interest.

## Footnotes

1 http://www.tenk.fi

## Supplementary Material

The files are available on Figshare, here is the link: https://doi.org/10.6084/m9.figshare.9638024.v1
